# Strain Specific Variations in *Acinetobacter baumannii* Complement Sensitivity

**DOI:** 10.3389/fimmu.2022.853690

**Published:** 2022-06-22

**Authors:** Gathoni Kamuyu, Giuseppe Ercoli, Elisa Ramos-Sevillano, Sam Willcocks, Chidchamai Kewcharoenwong, Pattarachai Kiratisin, Peter W. Taylor, Brendan W. Wren, Ganjana Lertmemongkolchai, Richard A. Stabler, Jeremy S. Brown

**Affiliations:** ^1^ Centre for Inflammation and Tissue Repair, UCL Respiratory, University College London, London, United Kingdom; ^2^ Department of Infection Biology, London School of Hygiene and Tropical Medicine, London, United Kingdom; ^3^ Department of Medical Technology, Faculty of Associated Medical Sciences, Chiang Mai University, Chiang Mai, Thailand; ^4^ Cellular and Molecular Immunology Unit, Centre for Research and Development of Medical Diagnostic Laboratories (CMDL), Faculty of Associated Medical Sciences, Khon Kaen University, Khon Kaen, Thailand; ^5^ Department of Microbiology, Faculty of Medicine Siriraj Hospital, Mahidol University, Bangkok, Thailand; ^6^ School of Pharmacy, University College London, London, United Kingdom

**Keywords:** complement resistance/sensitivity, *Acinetobacter baumanniia*, multi-drug resistance (MDR), virulence, Gram negative bacteria

## Abstract

The complement system is required for innate immunity against *Acinetobacter baumannii*, an important cause of antibiotic resistant systemic infections. *A. baumannii* strains differ in their susceptibility to the membrane attack complex (MAC) formed from terminal complement pathway proteins, but the reasons for this variation remain poorly understood. We have characterized in detail the complement sensitivity phenotypes of nine *A. baumannii* clinical strains and some of the factors that might influence differences between strains. Using *A. baumannii* laboratory strains and flow cytometry assays, we first reconfirmed that both opsonization with the complement proteins C3b/iC3b and MAC formation were inhibited by the capsule. There were marked differences in C3b/iC3b and MAC binding between the nine clinical *A. baumannii* strains, but this variation was partially independent of capsule composition or size. Opsonization with C3b/iC3b improved neutrophil phagocytosis of most strains. Importantly, although C3b/iC3b binding and MAC formation on the bacterial surface correlated closely, MAC formation did not correlate with variations between *A. baumannii* strains in their levels of serum resistance. Genomic analysis identified only limited differences between strains in the distribution of genes required for serum resistance, but RNAseq data identified three complement-resistance genes that were differentially regulated between a MAC resistant and two MAC intermediate resistant strains when cultured in serum. These data demonstrate that clinical *A. baumannii* strains vary in their sensitivity to different aspects of the complement system, and that the serum resistance phenotype was influenced by factors in addition to the amount of MAC forming on the bacterial surface.

## Introduction

An important component of the innate immune response to bacterial pathogens is the complement system. The human complement system consists of over 30 proteins either circulating in plasma or bound to cell surfaces and has roles in controlling infection by blood-borne pathogens, clearance of immune complexes, and in linking innate and adaptive immune responses (1–3). There are three complement activation pathways termed the classical (CP), lectin (LP) and alternative pathways (AP) that converge to cleave the central protein C3 to form the opsonin C3b, which then covalently binds to the pathogen’s surface. The CP is initiated by recognition of a bacterial pathogen by specific and natural antibody, serum amyloid P, or direct binding of the complement protein C1q, the first component of the CP pathway. The LP is activated by the recognition of various terminal sugars on bacterial surfaces by mannose-binding lectin (MBL), ficolins or collectin-11. The AP can be directly activated by pathogens and serves as an amplification loop of C3b bound to the pathogen surface. Complement activation results in the deposition of the opsonin C3b and iC3b on a pathogen’s surface which enhances phagocytosis by macrophages and neutrophils, as well as activation of the terminal complement pathway (complement proteins C5 to C9) to form a pore in bacterial surfaces termed the membrane attack complex (MAC). MAC causes bacterial lysis and in general is highly effective at killing Gram-negative bacteria, although some pathogens resist MAC-mediated killing and this is termed serum resistance (4). For some important bacterial pathogens significant variations in susceptibility to complement have been described between clinical isolates. For example, *Streptococcus pneumoniae* complement resistance varies markedly between clinical isolates and this is dependent on both the capsular serotype and non-capsular genetic variation (5–8). Similarly, *Klebsiella pneumoniae* strains vary in their degree of resistance to complement and this is influenced by capsule type and size, the presence or surface accessibility of the O-antigen in LPS (9, 10) and several additional genes identified through functional genomic studies (11). Importantly, for *S. pneumoniae* the invasive potential of different capsular serotypes inversely correlated with levels of opsonization with C3b/iC3b, demonstrating that differences in sensitivity to complement between strains of a bacterial pathogen can have important clinical implications (12).

The Gram-negative bacteria *Acinetobacter baumannii* is an increasingly common cause of nosocomial infections (particularly within intensive care units) that are often highly resistant to antibiotics and have a high mortality rate (13–17). *A. baumannii* virulence has been linked to serum resistance (18–22), and clinical *A. baumannii* strains vary in their degree of serum resistance (21, 23–27). Hence, the factors that cause differences in susceptibility to complement are likely to affect the virulence potential of *A. baumannii* strains. Similar to *S. pneumoniae* and *K. pneumoniae* the *A. baumannii* capsule protects the bacterium from complement-mediated immunity (19, 21, 22, 25, 28, 29). Variations in serum resistance between *A. baumannii* strains has been linked to capsule size (30), and specific mutations linking complement sensitivity to the chemical composition or size of the *A. baumannii* capsule have been identified (21, 31). Hence, the capsule is likely to be an important factor influencing variations between *A. baumannii* strains in sensitivity to complement mediated immunity. In addition, multiple proteins have been described that contribute to *A. baumannii* complement resistance (25, 32–35), suggesting that capsule independent mechanisms could also influence variations between strains in their degree of serum resistance.

Here, we have characterized in detail the complement sensitivity phenotypes of nine diverse *A. baumannii* clinical strains representing five different capsular loci (KL) types. We have used flow cytometry assays to compare deposition of C3b/iC3b and MAC formation (C5b-8/C5b-9) on bacterial surfaces. To characterize the functional consequences of complement activation we have measured complement-dependent neutrophil phagocytosis, serum resistance, and used genomic and RNAseq data to try and identify potential mechanisms underpinning differences between strains.

## Results

### The *A. baumannii* Capsule Inhibits Opsonization With C3b/iC3b and Protects Against MAC-Mediated Lysis

**Figure 1:**
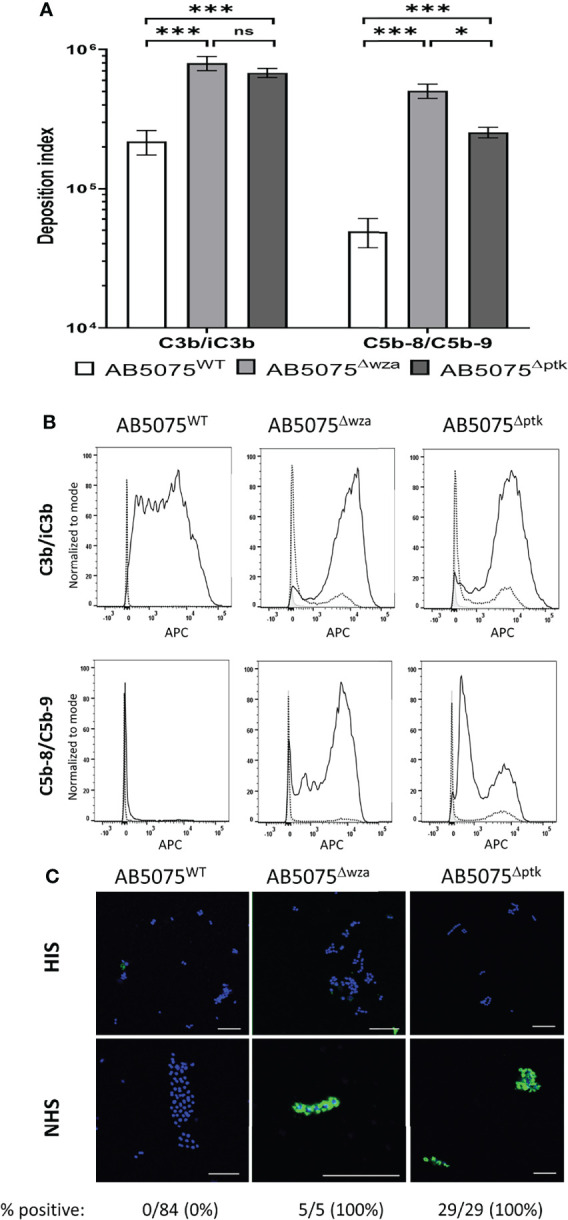
C3b/iC3b and C5b-8/C5b-9 deposition on the surface of wild-type and unencapsulated AB5075 isolates. 10^6^ CFU of bacteria was incubated with 25% normal human sera followed by incubation with either the mouse monoclonal antibody 6C9 (Millipore) or aE11 (Abcam) and detected using an anti-mouse IgG conjugated to either allophycocyanin (APC) (Jackson Immunoresearch) **(A)** Bar graph showing the deposition index calculated by multiplying the percentage of bacteria staining positive for the respective complement factor and the median fluorescent intensity of the positive population. Bacteria incubated with PBS only followed by the primary and secondary antibody was used to determine the negative population. **(B)** Representative histograms showing the deposition of C3b/iC3b (top panel) and C5b-8/C5b-9 (bottom panel) on 10^6^ CFU of the AB5075^WT^, AB5075^Δwza^ and AB5075^Δptk^ strains. Each graph shows a grey histogram that represents bacteria labelled with primary and secondary antibody only, while the black dotted and solid line represent bacteria incubated with heat-inactivated or normal human sera respectively. **(C)** Confocal imaging showing C5b-8/C5b-9 deposition on wild-type and unencapsulated AB5075^Δwza^ and AB5075^Δptk^ isolates following incubation with heat-inactivated sera (HIS) (top panel) or normal human sera (NHS) (bottom panel). Blue represent DAPI stained bacteria and green shows bacteria positive for MAC deposition. Figures below each panel represent the percentage of bacterial cells in each panel showing atleast some green fluorescent staining representing MAC deposition. A scale bar equivalent to 10 µm is indicated by a white line in all images. Bars represent mean values for each condition/strain and the error bars indicate standard deviations (SDs) (n = 3). T-test was used for statistical analysis *: p-value < 0.05, ***: p-value < 0.001, ns: p-value > 0.05. Representative data from two independent experiments is shown.

**Figure 2:**
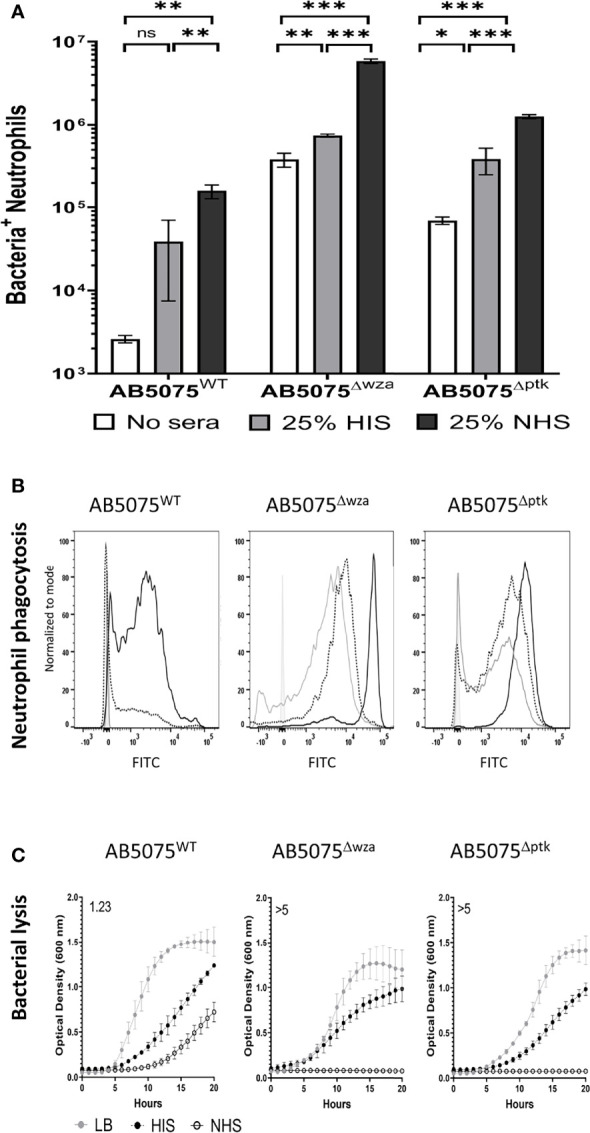
Human complement enhances neutrophil phagocytosis of wild-type and unencapsulated AB5075 isolates. FAMSE labelled encapsulated AB5075^WT^ and unencapsulated AB5075^Δwza^ and AB5075^Δptk^ strains were opsonized with either 25% heat-inactivated human sera, 25% normal human sera or unopsonised (PBS only) and incubated with healthy human neutrophils at Bacteria: Neutrophil MOI of 100:1. **(A)** Shows the percentage of FAMSE^+ve^ Neutrophils multiplied by the MFI of FAMSE^+ve^ Neutrophils [Mean (± standard deviation)] on the Y axis. Phagocytosis indices in unopsonised bacteria or those incubated with heat-inactivated sera, or normal sera for each isolate is represented in the white, grey and black bars respectively. **(B)** Representative histograms showing phagocytosis of FAMSE labelled *A. baumannii* isolates incubated with either normal sera, heat-inactivated sera, or PBS only. Each graph shows a solid grey histogram that represents neutrophils only (no bacteria), open grey histogram represents phagocytosis of unopsonised bacteria, black-dotted line represents phagocytosis of HIS opsonized bacteria and black solid filled histogram line represent bacteria opsonized with NHS. **(C)** Growth curves showing bacterial OD_600nm_ measurements over 24 hours obtained from bacteria incubated with either LB broth (grey circle), heat-inactivated sera (black circles) or normal human sera (open circle). Numbers on the top left corner indicate the doubling time ratio. The doubling time for each condition/strain was calculated to compare the growth rates of the three strains. Lower doubling times were observed in AB5075^WT^ compared to either AB5075^Δwza^ or AB5075^Δptk^ in LB and HIS. Similarly, lower doubling times were observed in AB5075^Δptk^ compared to AB5075^Δwza^. The lack of detectable bacterial growth in NHS for AB5075^Δwza^ and AB5075^Δptk^ prevented comparisons between strains analyses condition. Bars represent mean values for each condition/strain and the error bars indicate standard deviations (SDs) (n=3). T-test was used for statistical analysis *: p-value < 0.05, **: p-value < 0.01, ***: p-value < 0.001, ns: p-value > 0.05. Representative data from three independent experiments is shown.

Transposon mutagenesis has shown that the *A. baumannii* capsule is a major virulence factor that protects bacteria from complement-mediated lysis when grown in human sera or ascites fluid (28). To characterize this phenotype further, we measured the deposition of C3b/iC3b and C5b-8/C5b-9 (representing MAC formation on the bacterial surface) on the surface of the live wild-type encapsulated laboratory AB5075^WT^ strain and the unencapsulated isogenic strains AB5075^Δwza^ and AB5075^Δptk^ using flow cytometry. The levels of deposition of C3b/iC3b and C5b-8/C5b-9 differed significantly between the encapsulated AB5075 and unencapsulated strains (**Figures 1A, B**). Significant levels of C3b/iC3b deposition were detected on all three strains but with significantly higher binding observed for both unencapsulated strains (**Figure 1B**, top panel). In contrast, high levels of C5b-8/C5b-9 deposition were only detected on the unencapsulated strains with only low levels seen on the encapsulated AB5075^WT^ strain (**Figures 1A, B**, 2^nd^ panel). The detection of high levels of C5b-8/C5b-9 only on the unencapsulated AB5075^Δwza^ strain compared to AB5075^WT^ was confirmed by immunofluorescence microscopy (**Figure 1C**).

### Effects of the *A. baumannii* Capsule on Complement-Mediated Neutrophil Phagocytosis and Bacterial Lysis

The functional consequences of variation in C3b/iC3b and C5b-8/C5b-9 deposition on encapsulated AB5075^WT^ wild-type and the unencapsulated AB5075^Δwza^ strain were investigated by correlating the data with susceptibility to neutrophil phagocytosis and MAC-mediated bacterial lysis respectively. Using a flow cytometry assay (7, 36) demonstrated that the encapsulated AB5075^WT^ wild-type strain showed significantly higher levels of neutrophil phagocytosis when opsonized with untreated normal human sera (NHS) compared to either unopsonised bacteria, or bacteria opsonized with serum pretreated with heat to inactivate complement activity (HIS) (**Figures 2A, B**). In contrast, the unencapsulated strains had high levels of susceptibility to neutrophil phagocytosis under all conditions, although phagocytosis was still increased when opsonized with NHS compared to HIS (**Figures 2A, B**).

Whether C5b-8/C5b-9 deposition was associated with bacterial lysis was assessed by monitoring growth of the AB5075^WT^ wild-type and the unencapsulated AB5075^Δwza^ and AB5075^Δptk^ strains in the presence of 50% NHS or 50% HIS. Growth of the AB5075^WT^ strain was evident in NHS, albeit at a slower rate compared to HIS (**Figure 2C**). In contrast, the unencapsulated strains failed to grow in NHS despite growing well in HIS, indicating high sensitivity to NHS (**Figure 2C** and **Figure S1**). These data reconfirm that the capsule inhibits complement recognition of *A. baumannii* by inhibiting both C3b/iC3b and C5b-8/C5b-9 deposition and consequently reduces both neutrophil phagocytosis and NHS-mediated bacterial lysis.

### Significant Variation in C3b/iC3b Deposition Between Thai Clinical *A. baumannii* Strains

**Figure 3:**
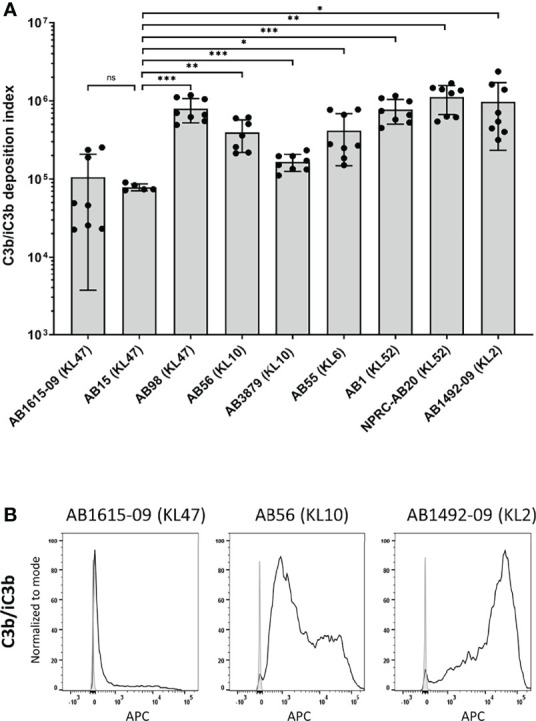
Complement component C3b/iC3b deposition on the surface of clinical *A. baumannii* isolates. 10^6^ CFU of bacteria was incubated with 25% normal human sera followed by incubation with the mouse monoclonal antibody 6C9 (Millipore) and detected using an anti-mouse IgG conjugated to allophycocyanin (APC) (Jackson Immunoresearch) **(A)** Bar graph showing the C3b deposition index computed by multiplying the percentage of bacteria staining positive for C3b/iC3b deposition and the median fluorescent intensity of the positive population (%Pos*MFI). **(B)** Representative histograms showing the deposition of C3b/iC3b on 1x10^6^ CFU of the three *A. baumannii* isolates. Each graph shows a grey histogram that represents bacteria labelled with primary and secondary antibody only and black solid line represent bacteria incubated with normal human sera. Bars represent mean values for each strain and the error bars indicate standard deviations (SDs) (n=8). T-test was used for statistical analysis *: p-value < 0.05, **: p-value < 0.01, ***: p-value < 0.001, ns: p-value > 0.05. Pooled data from three independent experiments is shown.

**Figure 4:**
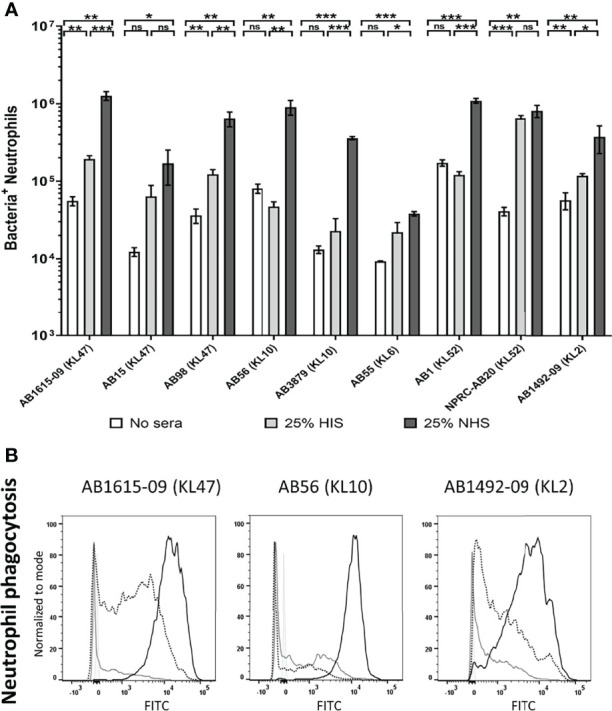
Human complement enhances neutrophil phagocytosis of clinical *A. baumanni* isolates. Nine FAMSE labelled *A. baumannii* isolates were opsonized with either 25% heat-inactivated human sera, 25% normal human sera or unopsonised (PBS only) and incubated with healthy human neutrophils at Bacteria: Neutrophil MOI of 200:1. **(A)** Shows the % of FAMSE^+ve^ Neutrophils * MFI of FAMSE^+ve^ Neutrophils [Mean (± standard deviation)] on the Y axis and the nine isolates with their capsule serotypes are indicated in brackets on the X-axis. Phagocytosis indices in unopsonised bacteria or those incubated with heat-inactivated sera, or normal sera for each isolate is represented in the white, grey, and black bars respectively. **(B)** Representative histograms showing phagocytosis of FAMSE labelled *A. baumannii* isolates incubated with either normal sera, heat-inactivated sera or PBS only. Each graph shows a solid grey histogram that represents neutrophils only (no bacteria), open grey histogram represents phagocytosis of unopsonised bacteria, black-dotted line represent phagocytosis of HIS opsonized bacteria and black solid filled histogram line represent bacteria opsonized with NS. Bars represent mean values for each condition/strain and the error bars indicate standard deviations (SDs) (n=3). T-test was used for statistical analysis *: p-value < 0.05, **: p-value < 0.01, ***: p-value < 0.001, ns: p-value > 0.05. Representative data from four independent experiments is shown.

To investigate potential variation in opsonisation with C3b/iC3b between genetically diverse clinical *A. baumannii* strains, the C3b deposition assay was repeated for nine *A. baumannii* clinical strains isolated from two hospitals in Thailand. All strains have been genome sequenced (30) and included different multilocus sequence typing (MLST) sequence types (ST) and capsule loci (KL) types. They comprised five ST2, three ST215 and one ST164 strains and three KL47, two KL10, two KL52, one KL6 and one KL2 capsular loci strains (**Table 1**) (36). Marked variation in C3b/iC3b deposition was identified between the clinical isolates (**Figures 3A, B**). Two strains (AB1615-09 and AB15, both KL47) had low levels of opsonization with C3b/iC3b whereas most strains (7/9 strains) showed significantly higher levels of binding to C3b/iC3b including the KL47 strain, AB98 (**Figures 3A, B**). Significantly higher levels of phagocytosis were observed when the *A. baumannii* strains were incubated in NHS (**Figures 4A, B**) compared to PBS. Susceptibility to phagocytosis was also improved by opsonization with HIS for four strains including two of the KL47 strains (AB1615-09 and AB98), one KL52 strain (NPRC-AB20), and the KL2 AB1492-09 strain, although to a lesser degree than after incubation in NHS with the exception of strain NPRC-AB20. These data show opsonization with C3b/iC3b in NHS compared to HIS improved neutrophil phagocytosis for seven of the *A. baumannii* strains investigated.

**Table 1:** *A. baumannii* clinical strains isolated from patients across either the Siriraj or Songklanagarind hospitals in Thailand [partially reproduced from Kamuyu et al, (36)].

Strains	KL class	MLST type	No. of cells evaluated (N)	Mean cell length (SD) (µM)	P-value (comparison with reference strain)
**Clinical *A. baumannii* strains**					
AB15	KL47	ST2	536	2.13 (0.44)	Reference strain
AB98	KL47	ST215	911	1.95 (0.43)	<0.0001
AB1615-09	KL47	ST164	449	2.28 (0.64)	<0.0001
AB56	KL10	ST2	427	2.03 (0.31)	0.001
AB3879	KL10	ST215	521	1.87 (0.22)	<0.0001
AB1	KL52	ST2	838	1.94 (0.29)	<0.0001
NPRC-AB20	KL52	ST215	626	2.61 (0.83)	<0.0001
AB55	KL6	ST2	1226	1.75 (0.23)	<0.0001
AB1492-09	KL2	ST2	427	1.81 (0.27)	<0.0001
**Laboratory *A. baumannii* strains**					
AB5075^WT^	KL25	ST1	639	2.46 (0.73)	Reference strain
AB5075^Δwza^	n/a		966	1.93 (0.40)	<0.0001

a Bacterial cell length was determined by confocal microscopy. ^b^n/a, not applicable.

### MAC Deposition on Clinical *A. baumannii* Strains Correlates With Opsonization With C3b/iC3b But Not With Degree of Serum Resistance

**Figure 5:**
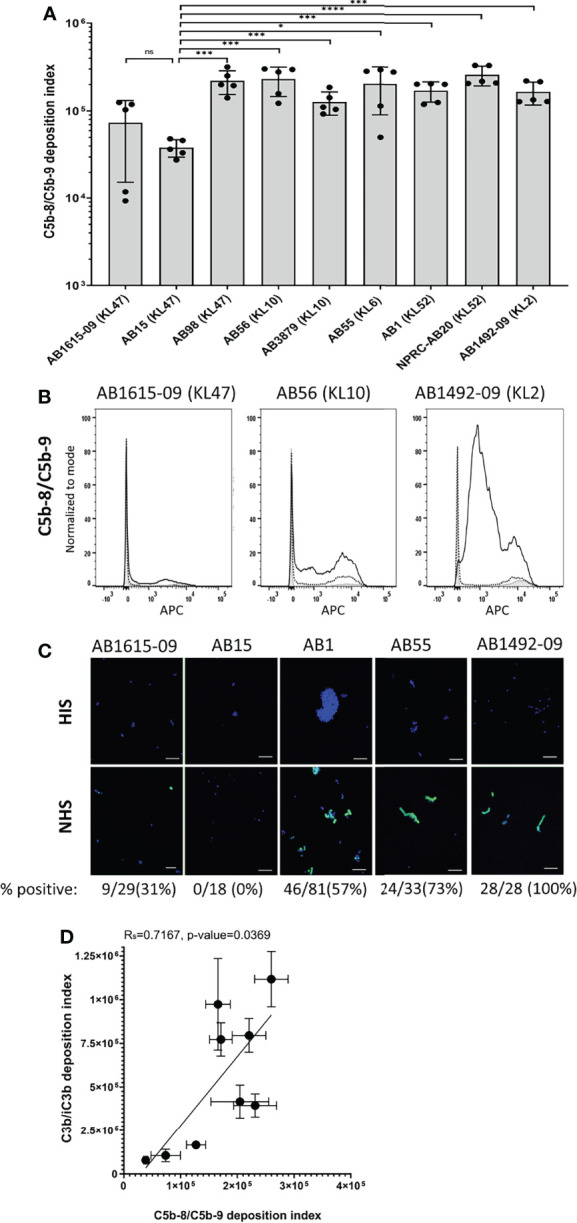
Membrane attack complex (C5b-8/C5b-9) on the surface of clinical *A. baumannii* isolates. 10^6^ CFU of bacteria was incubated with either 25% normal human sera or 25% heat-inactivated human sera, followed by incubation with the mouse monoclonal antibody aE11 (Abcam) that recognizes a neo-epitope on polymeric C9, and detected using an anti-mouse IgG conjugated to allophycocyanin (APC) (Jackson Immunoresearch). **(A)** Bar graph showing the C5b-8/C5b-9 deposition index (% positive * Median fluorescent intensity of bacteria staining positive for C5b-8/C5b-9 deposition). **(B)** Representative histograms showing the deposition of C5b-8/C5b-9 on 1x10^6^ CFU of three *A. baumannii* isolates. Each graph shows a grey histogram that represents bacteria labelled with primary and secondary antibody only, black-dotted line represents bacteria incubated with heat-inactivated human sera and black solid line represent bacteria incubated with normal human sera. **(C)** Confocal imaging showing C5b-8/C5b-9 deposition on a subset of *A.baumannii* clinical isolates following incubation with heat-inactivated sera (top panel) or normal sera (bottom panel). Blue represent DAPI stained bacteria and green shows bacteria positive for MAC deposition. Figures below each panel represent the percentage of bacterial cells in each panel showing atleast some green fluorescent staining representing MAC deposition. A scale bar equivalent to 10 µm is indicated by a white line in all images. **(D)** Correlation between C5b-8/C5b-9 deposition index and C3b/iC3b deposition on the nine clinical *A. baumannii* isolates. Bars represent mean values for each strain and the error bars indicate standard deviations (SDs) (n = 5). T-test was used for statistical analysis *: p-value < 0.05, ***: p-value < 0.001, ns: p-value > 0.05. Pooled data from two independent experiments is shown.

**Figure 6:**
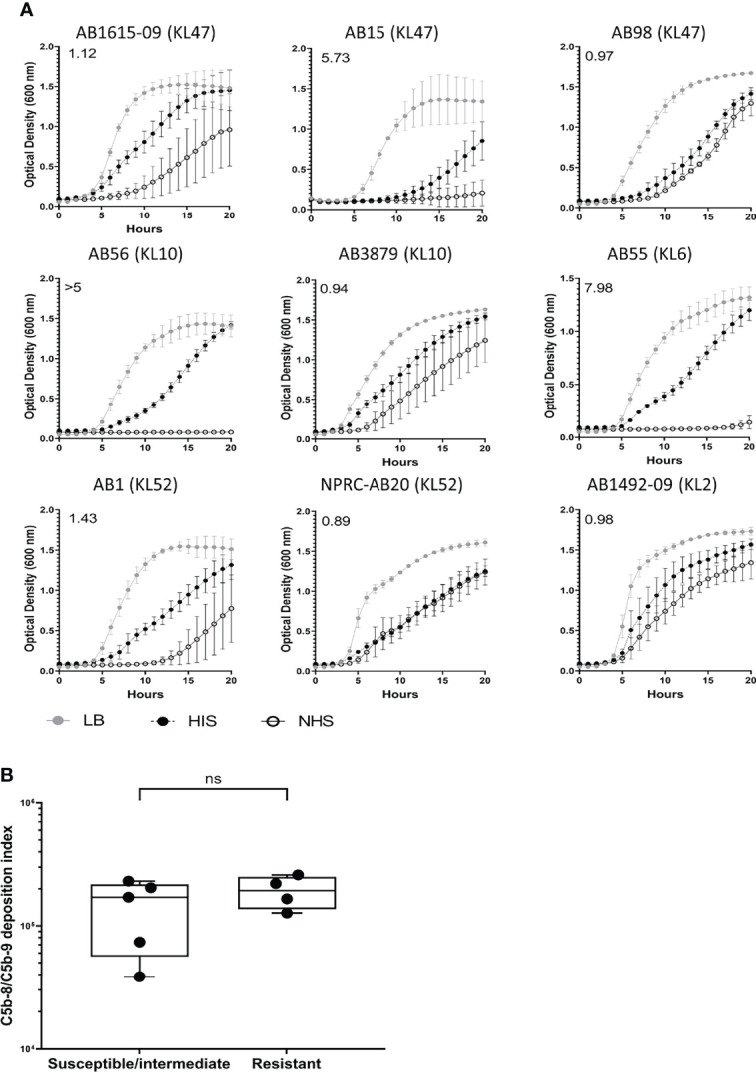
Clinical *A. baumannii* isolates predominantly show resistant or delayed sensitivity to human complement. 10^6^ CFU of bacteria was incubated with 50% normal sera or 50% heat-inactivated sera and the bacterial growth monitored over 24 hours with OD_600nm_ measurements obtained every 30 minutes. **(A)** Growth curves showing bacterial OD600nm measurements over 24 hours obtained from bacteria incubated with either LB broth (grey circle), heat-inactivated sera (black circles) or normal human sera (open circle). Numbers on the top left corner indicate the doubling time ratio. **(B)** Box and whisker graph comparing C5b-8/C5b-9 deposition levels in isolates classified as either susceptible, intermediate, or resistant to complement lysis. Line graphs indicate the mean value, and the error bars indicate standard deviations (SDs) (n = 9). Pooled data from three independent experiments is shown. ns: p-value > 0.05.

C5b-8/C5b-9 deposition on the bacterial surface was assessed using flow cytometry for the nine Thai *A. baumannii* strains and showed significant variations between strains (**Figure 5A**). On average, the lowest levels of C5b-8/C5b-9 deposition were detected on the AB15 (KL47) and AB1615-09 (KL47) strains. The highest levels were detected on the AB98 (KL47), AB56 (KL10), AB55 (KL6) and NPRC-AB20 (KL52) strains, with no significant differences in C5b-8/C5b-9 deposition detected between these four strains (P-value > 0.05, unpaired T-tests) (**Figures 5A, B**). Immunofluorescence assays to visualize C5b-8/C5b-9 deposition on a subset of the *A. baumannii* strains showed comparable results to the flow cytometry data, with strains having low levels of MAC detected by flow cytometry also showing the lowest proportion of bacteria associated with MAC by microscopy (eg strain AB15) (**Figure 5C**). The levels of C5b-8/C5b-9 deposition correlated positively with C3b/iC3b deposition (**Figure 5D**). To assess the relationship between C5b-8/C5b-9 deposition and serum resistance, the growth of all nine strains was monitored in the presence of NHS, HIS or nutrient rich media (LB). All strains showed reduced bacterial growth in both NHS and HIS compared to LB (**Figure 6**). The ratio of strain doubling time in NHS compared to that in HIS and the duration of lag phase was used to assess complement-dependent effects on growth and therefore the degree of serum resistance. Three broad patterns of susceptibility to complement-dependent growth inhibition were observed; strains AB56 (KL10), AB55 (KL6) and AB15 (KL47) that were highly susceptible to complement-dependent growth inhibition with doubling time ratios in NHS compared to HIS of greater than 5; strains AB1 (KL52) and AB1615-09 (KL47) had an intermediate susceptibility with doubling times in NHS compared to HIS greater than 1.0 and a lag phase duration of greater than 1 hour when cultured in NHS; and the strains AB98 (KL47), AB3879 (KL10), NPRC-AB20 (KL52) and AB1492-09 (KL2) which were resistant to complement-dependent growth inhibition with doubling times in NHS compared to HIS of less than 1.0 and no differences in the lag phase duration between NHS and HIS (**Figure 6A**). These data confirm significant variation in sensitivity to NHS-mediated bacterial lysis between the clinical strains investigated. Surprisingly, the levels of C5b-8/C5b-9 deposition between NHS-resistant and the other strains did not differ significantly (**Figure 6B**), indicating factors independent of the amount of MAC formation on the bacterial surface also influenced variations in serum resistance between strains.

### Capsule Size and Variation in *A. baumannii* Susceptibility to Complement

**Figure 7:**
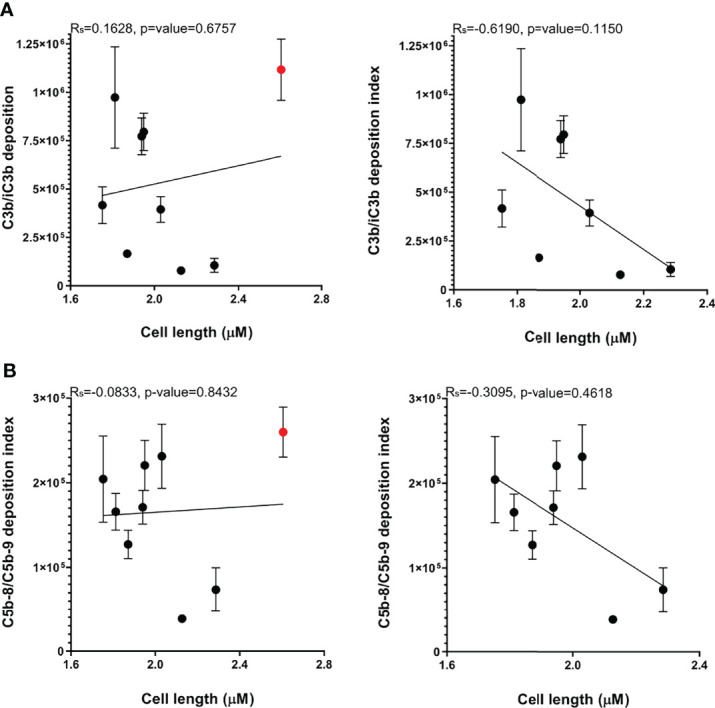
Bacterial cell length varies between *A. baumannii* clinical strains but does not correlate with C3b/iC3b, or C5b-8/C5b-9 deposition. The cell length measurements of a minimum of 400 representative bacteria of the nine *A. baumannii* strains were determined. **(A)** Correlation between median cell length and C3b/iC3b deposition in the nine clinical strains (left panel) or excluding the NPRC-AB20 strain (right panel) **(B)** Correlation between median cell length and C5b-8/C5b-9 deposition in the nine clinical strains (left panel) or excluding the NPRC-AB20 strain (right panel).

Published data demonstrated that the capsule contributes strongly to *A. baumannii* serum resistance, and our data obtained with the unencapsulated AB5075 strains demonstrates the capsule inhibits both C3b/iC3b and C5b-8/C5b-9 deposition on the bacterial surface. However, the large differences in the levels of C3b/iC3b deposition data between the three KL47 strains investigated here (**Figure 3A**) showed that KL type was not consistently associated with a specific complement sensitivity phenotype. We therefore investigated whether capsule size, independent of KL type, could influence *A. baumannii* sensitivity to complement. Cell length measured by microscopy was used as a proxy measurement of capsule size and was significantly reduced for the unencapsulated AB5075^Δwza^ strain compared to the encapsulated parental strain AB5075 (**Table 1**). Bacterial cell length also varied significantly between the nine clinical *A. baumannii* strains (**Table 1**). The two strains, that had the lowest C3b/iC3b and C5b-8/C5b-9 deposition levels, AB1615-09 and AB15, also had a larger cell length than many of the clinical strains. However, two strains of similar size to AB1615-09 and AB15 were relatively susceptible to complement recognition (AB56 and NPRC-AB20). Furthermore, cell length did not show a statistically significant correlation to levels of either C3b/iC3b or C5b-8/C5b-9 deposition even after exclusion from analyses of strain NPRC-AB20 that was a clear outlier (**Figures 7A, B** respectively).

### Genome Analysis of Genes Potentially Affecting Variations in Complement Sensitivity Between *A. baumannii* Strains

The lack of clear correlation with cell size indicated other factors to the capsule may also influence the variation in complement sensitivity between clinical *A. baumannii* strains. Hence, we used the available genome sequences to assess the distribution of genes that the published data indicate are required for *A. baumannii* serum resistance (25, 32, 33, 35) amongst the nine strains used in this study as well as a total of 220 Thai clinical isolates strains (30). Most of the genes (42/52 (81%) had a high degree of conservation at the amino acid level (90%+) and were present in the large majority of Thai strains (95%+, **Table 2**). Of the ten remaining genes, four had no identifiable equivalent in the Thai strain used for the BLASTP analysis (AB1615-09), and six had varying levels of amino acid conservation and were found only in a minority of the Thai strains analyzed (**Table 2**). This latter group could potentially be contributing towards differences in complement sensitivity between *A. baumannii* strains, and five of these are thought to be involved in capsule synthesis (ABUW_1868, ABUW_3828, ABUW_3830, ABUW_3831 and ABUW_3832).

**Table 2:** Genome data on the distribution of complement resistance genes amongst 220 Thai clinical *A. baumannii* strains (30).

Complement resistance gene ID	Putative gene function (annotation from ABUM genome)	% conservation (amino acid) ABUW versus AB1619-09^2,3^	Presence in the nine phenotyped Thai strains	Present in N (%) of total *A. baumannii* strains sequenced
ABUW_1868^1^	Hypothetical protein	98	1/9	34 (15.5)
ABUW_2898	Hypothetical protein	90	4/9	114 (51.8)
ABUW_3828^1^	Hypothetical protein	21	3/9	23 (10.5)
ABUW_3830^1^	UDP-glucose/GDP-mannose dehydrogenase	91	3/9	23 (10.5)
ABUW_3831^1^	Polysaccharide export protein	63	3/9	34 (15.5)
ABUW_3832^1^	Protein-tyrosine-phosphatase	87	3/9	23 (10.5)
ABUW_0383	Toluene tolerance efflux ABC transporter	96	9/9	220 (100)
ABUW_0384	Toluene tolerance efflux ABC transporter	90	9/9	215 (97.7)
ABUW_2168	Hypothetical protein	90	9/9	212 (96.4)
ABUW_2418	Lysine exporter protein	98	9/9	219 (99.5)
ABUW_3448	Glycosyltransferase	98	9/9	220 (100)
ABUW_3408	TPR repeat-containing SEL1 subfamily	92	9/9	220 (100)
ABUW_3829	UDP-N-acetyl glucosamine-2-epimerase	41	9/9	215 (97.7)
Highly conserved genes (n = 35)^4^		99-100	9/9	199-220 (90-100)

^1^involved in capsule synthesis.

^2^% match between ABUW-self BLASTP and the best match within ABUW-AB1615-09.

^3^ABUW complement evasion genes ABUW_2662, ABUW_3822, ABUW_3824, and ABUW_3825 have no strong match within theAB1615-09 genome.

^4^Highly conserved genes within Thai strains linked to complement evasion.

### Transcriptome Analysis of Complement Resistance Genes for Three *A. baumannii* Strains

**Figure 8:**
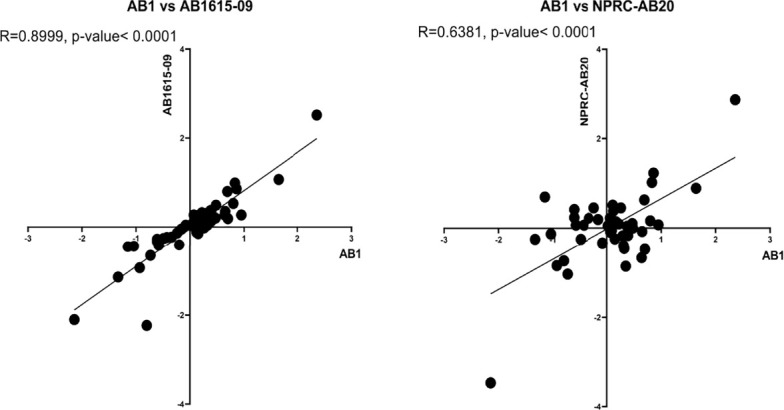
Complement resistance gene transcription levels correlate between phenotypically similar *A. baumannii* clinical strains. The relative expression levels of 52 complement resistance genes was determined in the complement resistant strain, NPRC-AB20 and two intermediate susceptible strains, AB1 and AB1615-09. Correlation between fold changes in expression of the 52 genes between the AB1 and AB1615-09 strain (left panel) and AB1 and the NPRC-AB20 strain (right panel).

To further assess non-capsule factors that could be influencing the differences in complement sensitivity between *A. baumannii* strains, we analyzed expression levels of the complement resistance genes for three *A. baumannii* clinical strains grown in either human sera or nutrient rich media. The strains selected for RNAseq analysis included two strains classified as having intermediate sensitivity to complement [AB1 (KL52) and AB1615-09 (KL47)] and one serum resistant strain with the same KL type as one of the intermediate strain [NPRC-AB20 (KL52)]. Direct comparison of the relative expression levels of each gene in human sera between strains demonstrated a strong correlation between the two intermediate serum resistance strains (R=0.90) (**Figure 8**, left panel), but poorer correlation for the serum resistant strain to either of the intermediate serum resistant strains (R=0.64 or 0.74) (**Figure 8**, right panel). This suggested expression levels of some serum resistance genes could reflect the differences in strain phenotype. To assess this, we investigated whether the effects of serum on expression of these genes differed between the serum resistant NPRC-AB20 (KL52) and the intermediate resistant strains. Using a cut-off value of log_2_ fold change of +/- 0.5, eight genes were identified that were either significantly upregulated or down-regulated when the NPRC-AB20 (KL52) strain was cultured in serum compared to broth (**Table 3**). Three of these genes showed differential expression in serum for the NPRC-AB20 (KL52) strain compared to AB1(KL52) and AB1615-09 (KL47) strains; ABUW_2637 which encodes a hypothetical protein, ABUW_3639 which encodes a putative response regulator, and ABUW_3822 which encodes a putative serine acetyltransferase (**Table 3**). These data indicate proteins encoded by these genes may have a role in variations in serum sensitivity between clinical *A. baumannii* strains.

**Table 3:** Transcriptome data for complement resistance genes showing genes with differential expression in human sera compared to nutrient rich media in the complement resistant NPRC-AB20 strain and the intermediate sensitivity AB1615-09 and AB1 strains.

Serum resistance genes	Putative gene function	Log_2_ Fold change in expression levels in NHS compared to broth					
		AB1615-09		AB1		NPRC-AB20	
		Fold change	P-value^1^	Fold change	P-value^1^	Fold change	P-value^1^
**Upregulated genes in the NPRC-AB20 strain**							
ABUW_2168	hypothetical protein	2.52	2.79E-12	2.36	5.70E-09	2.87	2.58E-05
***ABUW_2637***	***Hypothetical protein***	***0.86***	***3.08E-11***	***0.86***	***0.000400***	***1.23***	***1.12E-10***
ABUW_0385	toluene tolerance efflux transporter	1.07	0.00231029	1.64	3.05E-09	0.89	0.0001013
ABUW_0729	uppP	0.09	0.64808354	0.11	0.6985674	0.51	0.0121933
**Downregulated genes in the NPRC-AB20 strain**							
ABUW_2456	Putative Hydroxymethylglutaryl-CoA lyase	-2.10	2.86E-52	-2.14	1.50E-14	-3.47	6.80E-30
***ABUW_3639***	***response regulator***	***0.22***	***0.48382633***	***0.35***	***0.304992***	***-0.85***	***0.000370***
ABUW_1759	Extracellular serine proteinase	-0.93	6.19E-12	-0.93	8.58E-10	-0.85	1.71E-06
***ABUW_3822***	***Serine acetyltransferase***	***0.27***	***0.3983097***	***0.64***	***5.84E-05***	***-0.66***	***0.000789***

^1^ P-value for expression in serum versus broth.

^2^Genes in bold are show differential relative expression levels between strain NPRC-AB20 and AB1615-09 and AB1.

## Discussion

Complement is a key component of innate immunity which can control bacterial numbers by promoting phagocytosis or by direct killing through formation of the MAC on bacterial surfaces. Previous publications have shown that *A. baumannii* is susceptible to MAC and that the level of susceptibility can vary between strains (21, 23–27). We have investigated in detail variations in complement sensitivity between a select group of clinical strains isolated from hospital patients in Thailand using flow cytometry assays of complement recognition by the opsonin C3b/iC3b and the terminal complement components C5b-8/C5b-9 in combination with functional assays of complement-dependent neutrophil phagocytosis and susceptibility to MAC-mediate bacterial lysis. The data demonstrated marked variation in C3b/iC3b and MAC binding between the nine clinical *A. baumannii* strains, which as expected was partially dependent on KL type. Complement opsonization improved neutrophil phagocytosis of most strains. Importantly, although C3b/iC3b binding and MAC formation on the bacterial surface correlated closely, MAC formation on the bacterial surface did not correlate with variations between *A. baumannii* strains in serum resistance. As complement resistance can influence *A. baumannii* virulence (18, 20–22, 31), our results have important clinical implications. Variations in susceptibility to different aspects of the complement system may explain why some *A. baumannii* strains are more likely to cause invasive disease, as we have previously described for another encapsulated pathogen, *S. pneumoniae* (12).

Bacterial polysaccharide capsules form a layer around pathogenic bacteria blocking access of host immune effectors, including the complement system. Hence, it is not surprising that the capsule is a key factor inhibiting complement recognition of *A. baumannii* (21, 22, 25, 28, 29). Both the size and chemical composition of the *A. baumannii* capsule cause variations in complement sensitivity between strains (21, 29, 30). Our data using the AB5075 strain and two unencapsulated isogenic derivatives reinforce these previous findings and show the capsule inhibits both opsonization of *A. baumannii* with C3b/iC3b and formation of the MAC on the bacterial surface. The capsule only partially prevented opsonization of *A. baumannii* with C3b/iC3b, with significant detectable levels of C3b/iC3b on the encapsulated strain which also improved neutrophil phagocytosis. In contrast the capsule almost completely prevented detection of components of the MAC on the bacterial surface using flow cytometry or immunofluorescence, whereas high levels of C5b-8/C5b-9 were detected on the unencapsulated strains. The high level of MAC formation on unencapsulated strains was reflected in the serum killing assay, with unencapsulated strains showing no growth in sera containing complement activity. Despite the lack of detectable C5b-8/C5b-9 on the encapsulated AB5075 strain, this strain still had some sensitivity to MAC as there was a partial reduction in growth in serum compared to heat inactivated serum. Probably the high sensitivity of *A. baumannii* to MAC means that even a low level of detectable C5b-8/C5b-9 can result in significant bacterial killing.

Comparison of the complement-dependent phenotypes of the clinical strains showed a reasonably strong correlation between opsonization with C3b/iC3b and detectable C5b-8/C5b-9a on the bacterial surface. As opsonization with C3b/iC3b results in activation of the terminal complement pathway this correlation between C3b/iC3b and detectable C5b-8/C5b-9a on the bacterial surface would be predicted unless some *A. baumannii* strains specifically inhibit the terminal complement pathways. Our results included data for two or three strains each of three different KL types, allowing us to assess the effect of capsule structure on C3b/iC3b and C5b-8/C5b-9 deposition on the bacterial surface. Although in general these data were similar for strains with the same KL type, one of the KL47 strains showed marked differences in detectable C3b/iC3b and C5b-8/C5b-9 on the bacterial surface compared to the two other KL47 strains. Furthermore, C3b/iC3b deposition and detectable C5b-8/C5b-9 on the bacterial surface did not correlate with capsule thickness. These data indicate that although the capsule has a key role in *A. baumannii* complement resistance, other factors than capsule chemical composition and thickness also affect recognition of the bacteria by complement. The functional assays of complement dependent phagocytosis and NHS-mediated growth inhibition provide further support for this conclusion; relative sensitivity to both complement-dependent phagocytosis and NHS-mediated growth inhibition varied markedly between some strains with the same KL type (e.g. the two KL52 strains AB1 and NPRC-AB20 for complement-dependent phagocytosis, and the two KL10 strains AB56 and AB3879 for growth in sera). These data suggest there is a major role for capsule independent factors influencing variations between *A. baumannii* strains in their sensitivity to different aspects of the complement system. In addition, the lack of correlation between detectable C5b-8/C5b-9 on the bacterial surface and NHS-mediated growth inhibition suggested that the sensitivity level of a given *A. baumannii* strain to MAC is dictated by a combination of both the degree of C5b-8/C5b-9 binding to the bacterial surface and strain sensitivity to the subsequent physiological disturbance caused by MAC formation. In addition, the role of heat-stable bactericidal factors present in human sera such as natural antibodies, defensins, lysozymes are likely to contribute to the lack of correlation between C5b-8/C5b-9 deposition and the NHS-mediated growth inhibition observed between the *A. baumanni* strains evaluated. Differences in sensitivity to physiological disturbances caused by MAC and to non-complement dependent serum mediated immunity perhaps explain why some strains (eg NPRC-AB20) show no differences in impairment of growth in NHS and HIS compared to LB, despite high levels of MAC deposition. Furthermore, the functional consequences of complement-recognition for neutrophil phagocytosis or growth in NHS is likely to vary between strains. For example, although strain AB1615-09 was relatively resistant to opsonization with C3b/iC3b, this still resulted in significant improvements in phagocytosis and growth inhibition of this strain in NHS compared to HIS.

As well as the capsule, a large number (50+) of *A. baumannii* genes encoding proteins have been shown to affect serum resistance and therefore susceptibility to MAC (25, 32–35), only a minority of which are predicted to be involved in capsule structure and thickness. This is compatible with our data indicating capsule-independent effects on complement sensitivity, and that additional pathways are involved in mediating serum resistance independent of simply blocking complement activation and MAC formation on the bacterial surface. Our genomic analysis looking at the distribution of 52 complement resistance genes demonstrated the majority (35) were present in the 220 Thai strains evaluated. Of the remaining 17 absent from the genomes of a proportion of strains or showing allelic variation in amino acid composition, five are probably involved in capsule synthesis, leaving 12 that could mediate capsule-independent effects on strain complement resistance phenotypes. Differential expression of complement-resistance genes under clinically relevant physiological conditions could also result in variations between strains in their complement phenotype. We therefore compared RNAseq data from *A. baumannii* cultured in sera for two partially complement-resistant and one highly complement-resistant strains. The results showed strikingly strong correlation of complement-resistance gene expression for the two partially complement-resistant strains even though they belong to different KL and ST types. RNAseq results for both these strains correlated less well with the RNAseq data for the complement resistant strain, even though this strain had the same KL type as one of the partially complement resistant strains. Three complement-resistance genes were identified showing significant differential regulation in human sera between the MAC resistant and MAC intermediate resistant strains. These were ABUW_2637 a hypothetical protein, ABUW_3639 which encodes a putative response regulator, and ABUW_3822 which encodes a putative serine acetyltransferase. ABUW_3822 is likely to be involved in capsule synthesis therefore could easily affect complement sensitivity, but how the other two genes could affect serum resistance is not known (25). The genome and RNAseq data identified genes that could be influencing variations in complement resistance between *A. baumannii* strains. Future RNAseq analyses of gene expression by *A. baumannii* strains in NHS compared to HIS, and in HIS compared to LB broth should help determine genes that are differentially expressed specifically in response to complement activity rather than in response to serum alone. Candidate genes for further investigation could also be identified by extending the number of strains investigated for their complement phenotype and gene expression in human sera to identify those genes that consistently segregate with the strain complement phenotype. However, at present the high antibiotic resistance levels of the Thai strains has prevented the development of targeted mutation methods needed to characterise in more detail how individual genes influence strain complement sensitivity.

## Materials and Methods

### Bacterial Strains and Culture Conditions

The nine clinical *A. baumannii* isolates were obtained from patients admitted to Songklanagarind and Siriraj Hospitals, Thailand (**Table 1**) (36). AB5075 wild-type and unencapsulated AB5075^-wza^ were obtained from the Manoil lab *A. baumannii* mutant library (https://www.gs.washington.edu/labs/manoil/baumanniii.htm). Bacteria were cultured at 37°C on LB plates or in LB broth to an optical density at 600nm of 0.8 (approximately 10^9^ CFU/ml) and stored at -80°C in 10% glycerol as single use aliquots. The cell length of each *A. baumannii* strain was determined using confocal microcopy as reported previously (36). Briefly, bacteria were visualized by FITC-exclusion (2000 kDa FITC-Dextran, Sigma) using a Zeiss LSM 880 confocal microscope with ZEN Black 2.3 software. Bacterial sizes were determined using Image J 1.53a with at least 1000 individual bacilli detected per strain.

### Flow-Cytometry Detection of C3b/iC3b and C5b-8/C5b-9 Deposition on the Bacterial Surface

C3b/iC3b and C5b-8/C5b-9 (MAC) deposition on *A. baumannii* bacterial surface was assessed using flow cytometry as previously described (6, 7, 12, 36–38). 10^6^ CFU of *A. baumannii* was incubated with either 25% (the optimized concentration for differentiating between strains) normal human sera (NHS) or heat-inactivated human sera (HIS) for 30 minutes at 37°C, washed twice with PBS, followed by incubation in either the mouse monoclonal antibody 6C9 (Millipore) or aE11 (Abcam), in triplicate and detected using goat anti-mouse IgG-allophycocyanin (APC) (Invitrogen). Samples were analyzed using a BD FACSVerse and data processed using FlowJo software for Windows (version 10). Markers for identifying bacteria positive for the deposition of complement proteins were set using bacteria incubated with PBS and then incubated with the secondary antibody. Two independent experiments were conducted using stocks cultured on separate days and stored as single-use aliquots.

### Confocal Microscopy Detection of C5b-8/C5b-9 Deposition on the Bacterial Surface

To allow complement deposition, 10^6^ CFU of *A. baumannii* was incubated with either 25% (the optimized concentration for differentiating between strains) normal human sera (NHS) or heat-inactivated human sera (HIS) for 30 minutes at 37°C, washed twice with PBS, followed by incubation for 30 min with mouse monoclonal antibody aE11 (Abcam). Samples were washed twice with PBS, incubated for 15 min with Alexafluor488-conjugated anti-mouse IgG (Invitrogen). Bacterial DNA was stained by adding 4’,6-diamidino-2-phenylindole (DAPI), and DAKO mounting media (Agilent) was added as an antifade agent before sealing the slides. Fluorescent images were acquired using an Olympus TIRF confocal microscope and Fluoview software (Olympus Lifesciences).

### Serum Resistance Assay

10^2^ CFU of *A. baumannii* was incubated in either 50% NHS or HIS in triplicate and bacterial growth (OD_600nm_) at 37°C in 5% C02 was monitored every hour over a 24-hour period using the Spark multimode microplate reader (TECAN). 10^4^ CFU of the encapsulated laboratory AB5075^WT^ and the unencapsulated isogenic strains AB5075^Δwza^ strain was incubated in either 50% NHS or HIS in triplicate and the viable bacterial counts determined by CFU counts after 14 hours of incubation. Pilot experiments identified a concentration of 50% NHS gave the greatest discrimination in results between strains.

### Neutrophil Opsonophagocytosis Assays

Phagocytosis was investigated using an established flow cytometry assay, neutrophils extracted from fresh human blood from healthy adult donors and fluorescent *A. baumannii* labeled with 6-carboxyfluorescein succinimidyl ester (FAMSE, Molecular Probes) (7, 36, 38). Bacteria was opsonized with either 25% NHS, 25% HIS or no human sera for 30 minutes at 37°C, followed by the addition of 10^5^ neutrophils to a final multiplicity of infection (MOI) of approximately 1:100. A minimum of 5000 cells were analyzed by flowcytometry using a BD FACSVerse and data processed using FlowJo software for Windows (version 10). To identify the percentage of neutrophils associated with bacteria, neutrophils that had not been incubated with bacteria was used as the negative control. In order to combine the percentage of bacteria associated neutrophils and the intensity of association, a fluorescence index was determined by multiplying the percentage of positive neutrophils by the geometric median fluorescence of the positive population. All isolates were tested in parallel on the same day with the same batch of bacteria and neutrophils for each independent experiment. Results were compared for an individual strain under different conditions; variations in the degree of FAMSE labelling between *A. baumannii* strains prevented the direct comparisons of phagocytosis data between strains.

### Genomic Analyses for the Presence of Complement Resistance Genes

Genes required for complement resistant were obtained from previous publications (25, 32, 33, 35). Gene conservation for the global and Thai isolates (including AB1615-09) was determined with Roary (39) using a protein BLAST identity of 95% and a core definition of 99 (30). Using the AB5075-UW strain (CP008706) as a reference, a protein-protein BLAST was performed to identify orthologues in AB1615-09. The AB1615-09 orthologue was used to identify conservation within the Thai *A. baumannii* strains described here.

### Transcriptome Analyses of the Relative Expression of Complement Resistance Genes in *A. baumannii* Strains Grown in Either Nutrient Rich Media or Normal Human Sera

Transcriptome analyses by RNAseq was determined as previously described (40). Briefly, bacterial RNA was extracted from three *A. baumannii* strains initially cultured in LB to an OD_600_ of 0.5-0.6, then transferred in triplicate, to either 50% normal human sera (NHS) or 50% fresh LB for 1 hour. The Mirvana RNA kit (Applied biosystems, Foster City, CA, USA) was used for RNA extraction, with an additional physical lysis step using 0.1 mm glass beads (MP Biomedicals, USA), treated with Turbo DNase (Applied biosystems, USA) and deleted of ribosomal RNA using Ribo-Zero Magnetic Kit Bacteria (Illumina, USA) before preparation of sequencing libraries using the KAPA RNA HyperPrep kit (Roche Diagnostics, Switzerland) and 8 cycles of amplification. Libraries were multiplexed to 24 samples per run and single-end sequenced with the NextSeq 500 desktop sequencer (Illumina, San Diego, CA, USA) using a 75-cycle high-output kit. Raw fastq data was parsed through Trimmomatic (leading 3, trailing 3, sliding window:4:20, minlen 36) to remove low quality bases. Resultant high-quality reads were mapped the AB1516 genome. Transcripts were quantified using Salmon (41) using validate mappings and 1000 bootstraps. Comparative analysis was performed using Sleuth. Raw RNAseq data was uploaded to the European Nucleotide Archive (ENA) and the individual data file accession numbers are as follows: ERR8982504, ERR8982505, ERR8982506, ERR8982507, ERR8982508, ERR8982509, ERR8982510, ERR8982511, ERR8982512, ERR8982513, ERR8982514, ERR8982515, ERR8982516, ERR8982517, ERR8982518, ERR8982519, ERR8982520, ERR8982521.

### Statistical Analysis

Statistical analyses were conducted using GraphPad Prism version 8 (GraphPad, USA). Data are presented as means, and the error bars represent standard deviations. Parametric data were analyzed using unpaired Student’s T test. Doubling times from growth curves were calculated using the log of exponential growth equation in GraphPad version 8 (https://www.graphpad.com/guides/prism/latest/curve-fitting/reg_log-of-exponential-growth.html). The optical density values obtained from bacterial growth at the exponential phase (6-16 hours) were log transformed and the non-linear regression of exponential growth with a log population equation used to calculate doubling time in the presence of respective sera. The doubling time ratio was calculated by dividing doubling time in respective normal human sera over doubling time in heat-inactivated sera (DT_NHS_/DT_HIS_).

## Data Availability Statement

The datasets presented in this study can be found in online repositories. The names of the repository/repositories and accession number(s) can be found below: ENA ERR8982504 - ERR8982521.

## Author Contributions

JSB, BWW, RAB and GL led the design and setup of the project. PK and PWT provided bacterial strains. GK, GE, ER-S, SW performed the experiments. RS performed the bioinformatics analysis. GK analysed the data. GK and JB wrote the manuscript. All authors contributed to the article and approved the submitted version.

## Funding

This work was undertaken at UCLH/UCL who received a proportion of funding from the Department of Health’s NIHR Biomedical Research Centre’s funding scheme and was also supported by an MRC DPFS MR/S004394/1 to RAS, BWW, GL, and JSB. The funders had no role in study design, data collection and interpretation, or the decision to submit the work for publication.

## Conflict of Interest

The authors declare that the research was conducted in the absence of any commercial or financial relationships that could be construed as a potential conflict of interest.

## Publisher’s Note

All claims expressed in this article are solely those of the authors and do not necessarily represent those of their affiliated organizations, or those of the publisher, the editors and the reviewers. Any product that may be evaluated in this article, or claim that may be made by its manufacturer, is not guaranteed or endorsed by the publisher.

## Supplementary Material

Supplementary Figure 1
*A. baumannii* capsule inhibits complement-mediated bacterial lysis in the laboratory AB5075 strain. 10^4^ CFU of the encapsulated laboratory AB5075^WT^ and the unencapsulated isogenic strains AB5075^Δwza^ strain was incubated in either 50% NHS or HIS in triplicate and the viable bacterial counts determined by CFU counts after 14 hours of incubation. Bars represent mean values for each condition/strain and the error bars indicate standard deviations (SDs) (n=3). T-test was used for statistical analysis *: p-value < 0.05, **: p-value < 0.01, ***: p-value < 0.001, ****: p-value <0.0001 ns: p-value > 0.05.
