# Supplementary figures and images for: Strain Specific Variations in Acinetobacter baumannii Complement Sensitivity
Source: Front Immunol. 2022 Jun 22;13:853690. doi: 10.3389/fimmu.2022.853690 (PMC9258041; doi:10.3389/fimmu.2022.853690)

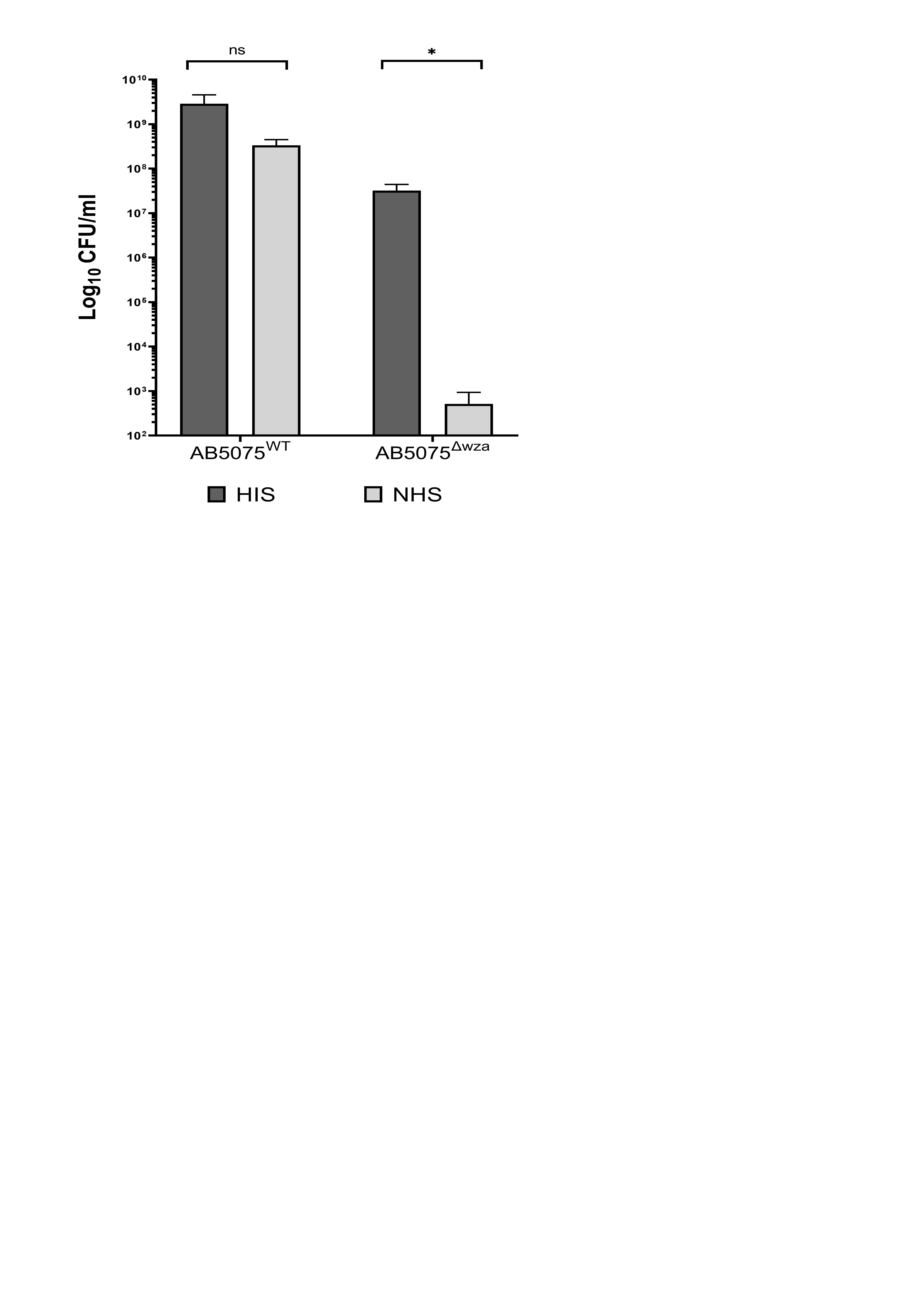

Supplement: Supplementary Figure 1 — A. baumannii capsule inhibits complement-mediated bacterial lysis in the laboratory AB5075 strain. 104 CFU of the encapsulated laboratory AB5075WT and the unencapsulated isogenic strains AB5075Δwza strain was incubated in either 50% NHS or HIS in triplicate and the viable bacterial counts determined by CFU counts after 14 hours of incubation. Bars represent mean values for each condition/strain and the error bars indicate standard deviations (SDs) (n=3). T-test was used for statistical analysis *: p-value < 0.05, **: p-value < 0.01, ***: p-value < 0.001, ****: p-value <0.0001 ns: p-value > 0.05. [file Image_1.jpeg]
